# New insights into improving acidic aluminum fuel cell for powering electrical vehicles

**DOI:** 10.1038/s41598-025-93496-2

**Published:** 2025-04-02

**Authors:** Mohammed L. Alazmi, Mai S. Alsubaie, Howida A. Fetouh

**Affiliations:** Chemistry Department, Faculty of Science, Alexandria University, P.O. Box 426, Alexandria, 21321 Egypt

**Keywords:** Aluminum, Fuel, Cell, Poison, Hydrogen, Evolution, Performance, Chemistry, Energy science and technology, Materials science

## Abstract

This study-involved novelty of high power new aluminum (Al) fuel cell in 0.1 M HCl (less corrosive and strong conductive electrolyte instead of the aggressive alkali). The performance of aluminum (Al) anode enhanced by using antimony sulphate Sb_2_(SO_4_)_3_ salt effective poison (in acidic solution) the parasitic hydrogen evolution reaction (HER). Antimony ad-atom specifically adsorbed at cathodic sites of Al surface and stopped HER *via* retardation of recombination of step of H_ads_, consequently the formation of molecular hydrogen and its release from aluminum surface. The production of sustainable electricity from AFC and maximum power density achieved by Sb adsorption and using the low cost prepared porous air cathode (sulphur-Fe_2_O_3_-doped NiO nanocomposie). Corrosion control of Al and using NiO air cathode improved cell discharge capacity. The best cell parameters obtained at 1 × 10^− 5^M Sb_2_(SO_4_)_3_ were (cell potential 2.4 V, current density 20.4 Am^− 2^ and electrical power 30.30 kwhkg^− 1^.

## Introduction

In the near future, transportation vehicles will negatively influenced as the global problem of energy scarcity will peaked due to the limited energy sources. Fueling vehicles by aluminum fuel cell (small size and long shelf life) is an argent solving strategies^1^. Electrons (es) produced from spontaneous anodic oxidation of Al equal the current flow from Al anode to the air cathode; ions migrate in electrolyte to oppositely charged electrodes^2^. Al/HCl fuel cell is a good alternate to Al/NaOH because of the rapid corrosion rate of aluminum^3–5^. Cell power of Al/HCl maximized by using an efficient electro catalyst for the slow oxygen reduction reaction (ORR) that causes cathode polarization that retarding the current flow. Different cathode materials tested to optimize the electrocatalyst for ORR.

AFC represented as Al/ 0.1 HCl/air O_2(g)_/Pt or graphite with solid electrolyte operates without the need to the expensive ion exchange membrane^6^. It has high energy density, flat discharging at ambient temperature, long shelf life if stored dry), ecofriendly and cheap. The cell capacity is independent on the load and the operating temperature^2,5^. However, the cell dryness limited the shelf life, output power and the operating temperature range.

In alkaline AFC, HER accelerated Al corrosion. The carbonation of air cathode (as white precipitates of CaCO_3_ and MgCO_3_ from hard water) is an obstacle^7^. The sustainable air (O_2(g)_) cathode elevated the theoretical specific energy of AFC. The electrochemical equivalent of Al is 2.98 (Ahg^-1^) and the theoretical power is 8.1 (Whg^-1^). The theoretical cell potential (E_Theoretical_ (2.7 V) and the maximum practical cell potential can be 1.4 V. The lower practical cell potential under load attributed to the polarization of air cathode by carbonate scales formed at high pH, other interference electrodes processes and HER at Al surface, Eq. 1^2,5,7^.

1 $${{\text{E}}_{{\text{cell}}}}_{{{\text{practical}}}}={\text{ }}{{\text{E}}_{{\text{cell thermodynamic}}}} - {\eta _{\text{a}}} - {\text{ l}}{\eta _{\text{c}}}{\text{l }} - {\text{IR}}$$

Where η_a_, η_c_ are the overvoltage for anodic and cathodic reactions respectively; IR is the Ohmic drop overvoltage across the cell due to the resistance of the solution, the electrical connection and the corrosion products.

During cell discharging, Al oxidized into Al(III) ion that hydrolyzed by water producing H_2(g)_ as represented in Eqs. 2, 3.

2 $${\text{Anodic discharge reaction}}:{\text{ Al}}\,+\,{\text{3}}{{\text{H}}_{\text{2}}}{\text{O }} \rightleftharpoons {\text{ Al}}{\left( {{\text{OH}}} \right)_{\text{3}}}+{\text{3}}/{\text{2 }}{{\text{H}}_{{\text{2}}({\text{g}})}}$$

3 $${\text{The cathodic discharge reaction}}:{\text{1}}/{\text{2 }}{{\text{O}}_{{\text{2}}({\text{g}})}}\,+\,{{\text{H}}_{\text{2}}}{{\text{O}}_{({\text{l}})}}\,+\,{\text{2}}{{\text{e}}^ - } \rightleftharpoons {\text{2O}}{{\text{H}}^ - }_{{({\text{aq}}.)}}$$

The theoretical specific discharging energy depends only on Al anode, while the practical energy limited by the polarization of air cathode. The parasitic HER and the slow ORR are the main problems^2^. The reduction of H^+^ at Al surface (self-discharging) degrades the columbic efficiency of Al anode. The overvoltage for hydrogen evolution reaction of Al surface is around 120 mV and the discharge step (H^+^+ e^-^ $\rightleftharpoons$ H_ads_.) is the rate-determining step. The rate of combination step (H_ads_. + H_ads_. $\rightleftharpoons$ H_2(g)_). On All these challenges changed the cell potential during discharging.

The polarization (decrease the closed E_cell_ sharply with increasing the discharging current (I) due to the diffusion and the limitations of oxygen air cathode. CO_2(g)_ absorption in porous cathode hindered ORR (by blocking the pores and prevent entrapment of air oxygen. The large size entrapped CO_2(g)_ causing mechanical damage or decrease cathode performance; H_2_O vaporization increases electrolyte concentration, as a result the cell dry out and short circuited. Spontaneously formed Al_2_O_3_ (surface oxide film protected Al surface against corrosion is stable in the neutral and many acid solutions but it is attacked by the alkali). Al is colorless, forms nontoxic corrosion products, electrically and thermally conductive, characterized by the lightweight and good mechanical strength^8^. However, HER and slow ORR limited the commercialization of AFC.

Thermodynamic corrosion tendency of Al in HCl is much less than in NaOH. Corrosion rate (CR) of Al controlled by the addition of small concentration of corrosion inhibitor [CI] for HER. Inhibition mechanism involved inhibition of: Al dissolution, HER or both reactions. Barrier surface CI forms molecular adsorbed insulating surface film. The CI is organic, inorganic substances; oxidizing or non-oxidizing compounds)^8^.

For alkaline AFC: discharge capacity of cell potential (E_cell_) with time increased from 162.46 mAhcm^− 2^ to 267.41 mAhcm^− 2^ on using 10 mM (gL^-1^) ZnSO_4_. The zinc ion sacrificially protected Al surface. The electrically conductive Al_2_O_3_ control cell performance^9,10^. AFC discharge *via* ionic migration of OH^-^ ions through Al_2_O_3_. Al dissolve at Al_2_O_3_ /electrolyte interface. In 2.0 M NaOH, AFC series stack gave open circuit potential (E_OCP_) only 6 V, P_max_.180.6 kWm^− 2^ at room temperature. The low energy density 1 Whg^-1^(Al) attributed to the slow ORR at ordinary Pt or graphite © cathode. Low cost AFC stack of constant 12 V is required for powering the electrical vehicle. Acidic AFC (Al/ 0.1 M HCl) rarely reported, so this study aims maximizing the electrical power of Al/HCl FC *via* stopping the parasitic HER and catalyzing the slow ORR.

## Experimental

All chemicals involved in this study were of analytical grade purity and used without further purification: Iron sulphate, nickel sulphate, 37% HCl purchased from Sigma Aldrich Co.

New AFC (anode: Al anode/air cathode) designed. The Ohmic resistance minimized by using thin Nafion membrane and good electrical conductors copper wires^11^.

The porous electrocatalyst for ORR prepared as NiO nanocomposite (NiONC) contained NiONPs dopped by sulphure and ferric hydroxide (Fe_2_O_3_). Following Thermal co-precipitation reaction^12,13^: 6.0 g FeSO_4_.7H_2_O + 18.4 g ferri-NH_4_-sulphate dissolved in 100 mL distilled water. The mixture heated at 45^o^C, agitated at 150 rpm for 1.0 h. pH 11 adjusted using 4.0 M NaOH. Then the solution continuously agitated at 50 rpm at 45^o^C for 5 h. Ferric oxide (Fe_2_O_3_) nanoparticles (NPs) separated and filtered using syringe filter paper, then washed by distilled water or acetone and calcined at 50^o^C until complete dryness. A weight 4.0 g CT swelled in 150 mL 20% acetic acid. 1.5 g Fe_2_O_3_NPs added to chitosan solution followed by agitation at 50 rpm, 45^o^C, and pH 11. Reaction mixture kept at 90^o^C for 2.0 h. Coated NPs separated by filtration and washed by distilled water. Further stability achieved by addition 10 mL 0.01 M epichlorohydrin crosslinker (ECH) and agitation at 45^o^C, pH 11. Fe_2_O_3_NPs coated by cross-linked chitosan recovered. NiONPs prepared and coated following the same procedure using 20.0 g NiSO_4_^12^.

**Fig. 1:**
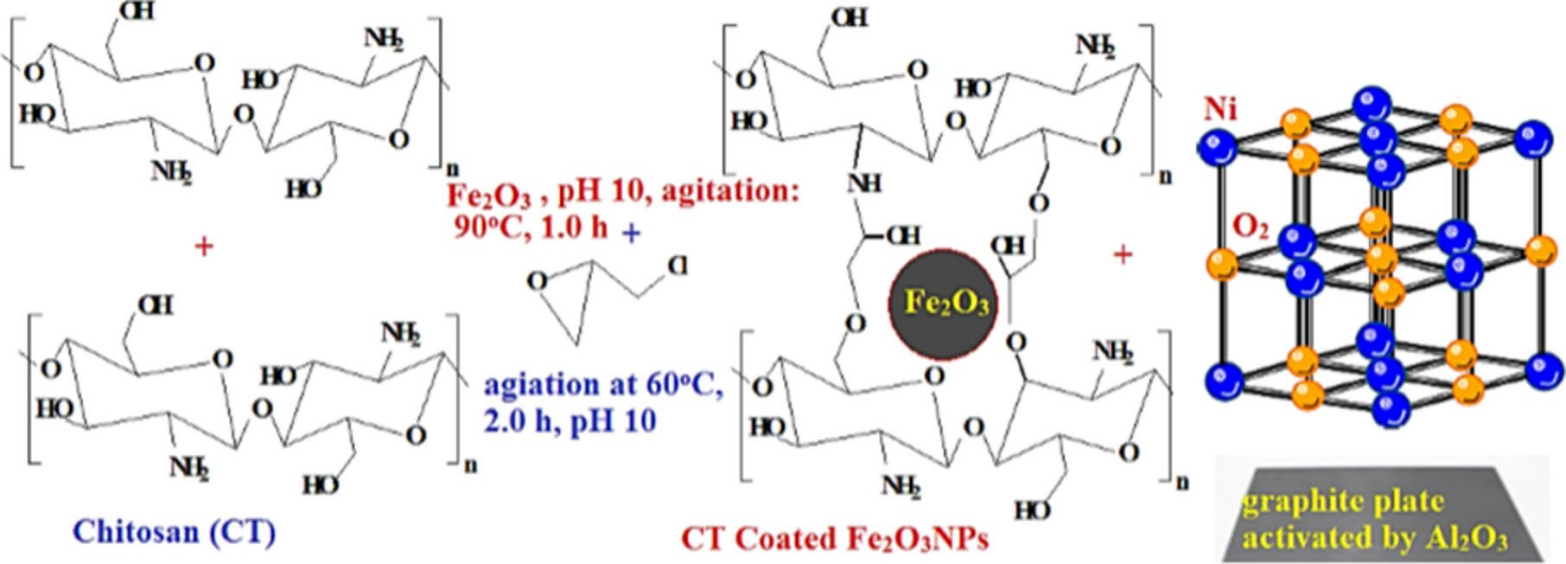
Schematic flowchart for fabrication air cathode.

The cathode catalyst (NiO) and the promoter (Fe_3_O_4_@CT) blended: 1.0 g Fe_3_O_4_ NPs: 3.0gNiO NPs + 10 mL 0.5 M sodium dodecyl sulphate (anionic surfactant) added with vigorous stirring at pH 11 (adjusted by 5.0 M NaOH), 3 mL cross linker added and solution refluxed for 1.0 h at 60^o^C. Catalyst composite spin coated on graphite C rod (activated by Al_2_O_3_-double distilled water paste). The cross sectional area 8 mm^2^ cleaned by acetone, dried by nitrogen, spin coated using spin coater (Sputter coating unit (JEOL Fine coat Ion Sputter, JFC-1100E, Japan) run at 2 × 10^3^ revolutions min^-1^ for 35 s.). The nanocomposite catalyst (S-NiO + Fe_2_O_3_ thin films (TF)) annealed at 200 °C for 2.0 h to achieve homogeneous improved film thicknesses. Cathode fabrication illustrated as shown in Fig. 1^12^.Air cathode left cooled to the room temperature and fabricated in small area for improving handling and storage.

Iron oxide is catalyst promoter, NiO is the active catalyst, and graphite (C) activated by Al_2_O_3_ is the solid support for the catalyst^14^.

Catalyst NiO sample characterized^12^. Crystallinity by powder XRD diffraction (pXRD) patterns at 25^o^C using Cu-K_α_ wavelength radiation (λ) 1.541 Å, 40 kV using Bruker D8 XRD-D2 Phaser (Germany) advance diffractometer. Angle range 5^o^ -70 ^o^, 0.02^o^ step/1^o^ min.^-1^. Surface morphology and particle size using field emission scanning electron microscope (FE-SEM) (SEM, JEOL SM-6510LV) accelerated at 20 kV voltage, gold-coated using EMITECH K550X sputter coater. Porosity using 99.99% pure N_2(g)_ adsorption-desorption isotherms at its boiling point 77 ^o^K after sample degassing in BELPREP- Vacuum II, BEL Japan, Inc. processor at 300^**°**^C for 3 h. Specific BET surface area (a_SBET_), total pore volume (V_T_) and mean pore diameter (D_P_) calculated using Brunauer-Emmett-Teller (BET) theory. The average pore radius calculated using Eq. 4.

4 $$\:\text{Average\, pore\, radius\, r\, (nm)}=\frac{{2}\check{a}V_{T}(ml/g)}{{a}_{S,BET}({m2/g)}^{\:}}x1000$$

**Fig. 2:**
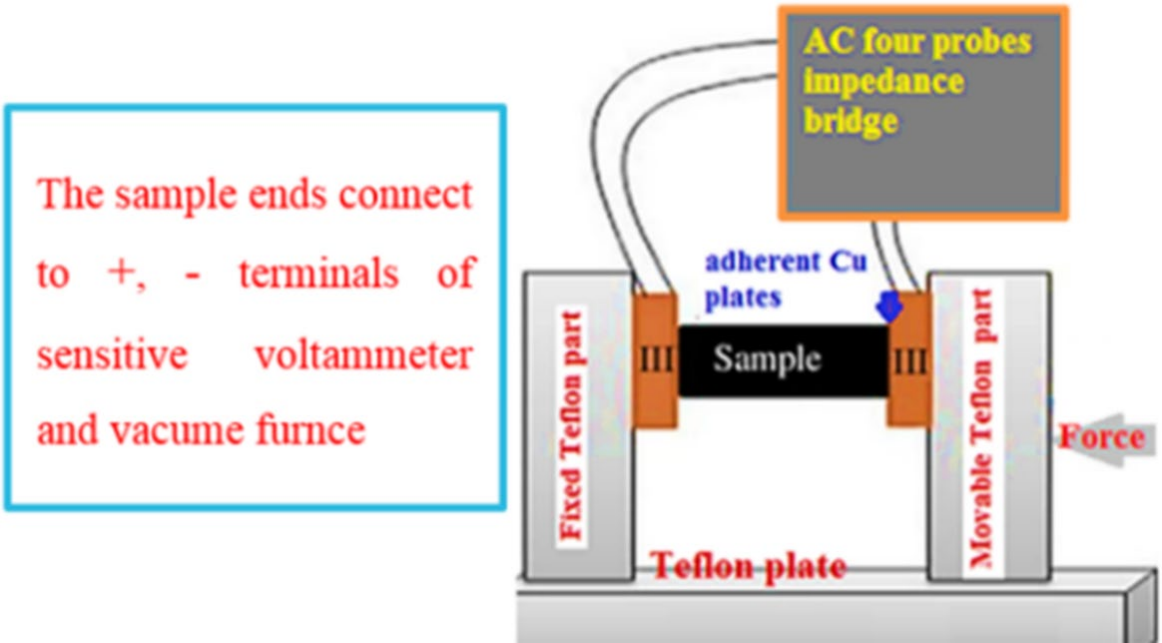
Impedance Bridge for measuring σ_AC_ at frequency range 100 Hz to 100 MHz).

Electrical conductivity (σ) measured at different temperature using four probes Agilent 4294 A Impedance Bridge-400 AC signal amplitude 10 mV. The experimental setup represented in Fig. 2. The test NiONC sample compressed at10 tons cm^− 2^ into a tablet of cross sectional area (a) 1.40 cm^2^, 2 mm thickness (d), holded between two Cu electrodes with conductive Ag paste and annealed at 60 °C, σAC calculated using Eq. 5^15^.

5 $$\sigma \,=\,{\text{I d }}/{\text{ V}}*{\text{ a}}$$

Where: I current (A) and V: potential drop across sample.

Current and voltage measured in triplicates. The average electrical conductivity calculated as (the reciprocal of the electric resistivity)^15^.

For 1.0 mL uniform diluted sample suspension in distilled water, Zeta (ζ) potential recorded using Zetasizer Nano ZS series (Malvern, ZEN 3600, Australia) placed in a cuvette fixed between two Pt electrodes. Particle size (PS) determined using photon correlation spectroscopy (PCS) using the same apparatus, non-invasive backlight scattering at 173° detection angle at 25.0 ± 0.1 °C. Both ζ and PS determined in triplicates for reproducibility. The surface acidity of NiONC catalyst determined by non-aqueous titration against *n*-butylamine base (pK_a_ 10.73). The electrode potential (mV) measured as a function of the progressive increase (mmol g^− 1^[base concentration]) to NiONC emulsion recorded^16^.

**Fig. 3:**
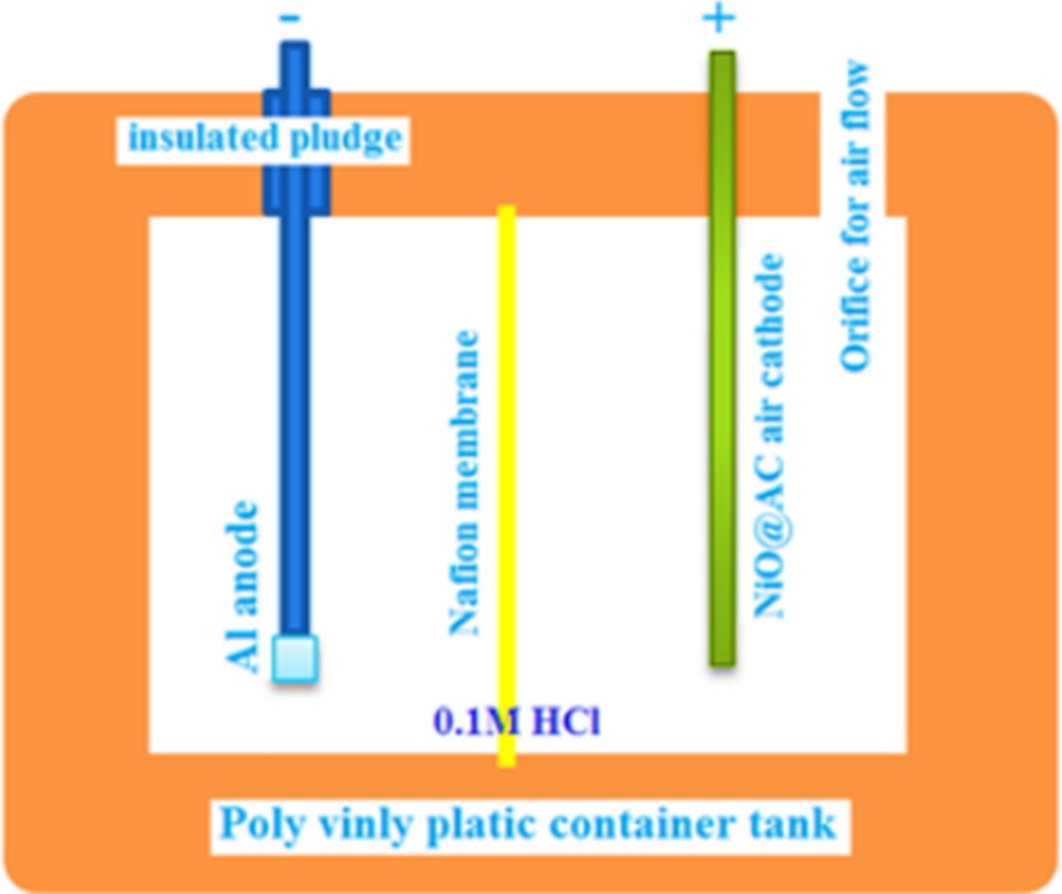
Lab scale design of AFC: A sufficient, high and current delivery capacity. De-aeration of anode chamber prevented electrons consumption by dissolved oxygen to allow electric current (liberated electrons) flow through the external circuit.

In a simple designed AFC represented in Fig. 3: A thin plate of commercial de-aerated Al anode (chemical composition showed in Table 1) connected by thin electrically conductive copper to the prepared air cathode (Pt/graphite/activated graphite spin coated by NiONC).

**Table 1:** Chemical composition of the commercial al anode.

Element	Si	Fe	Cu	Cd	Al
Weight% (Wt. %)	0.088	0.150	0.009	0.002	99.749

The Nafion membrane is chemically inert sulfonated tetrafluoroethylene (perfluorinated) terpolymer (Merck-Sigma Aldrich).

**Fig. 4:**
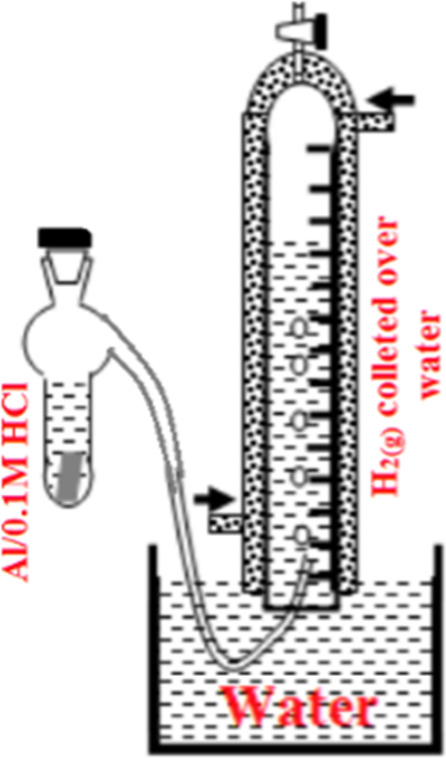
Experimental set up for gasometry method used for monitoring HER.

The plate Al sample (0.5 cm width x5.0 cm) length immersed in 0.1 M HCl in the absence and the presence of different [Sb_2_(SO_4_)_3_].Volume of H_2(g)_ evolved recorded as a function of time using chemical gasometry method represented in Fig. 4. HER monitored by water displacement. Reaction rates taken as equals to the slope of the obtained straight line^17^.

The two-steps simultaneous H_2_O reduction represented by Eqs. 6, 7^18^.

6 $${\text{A}}{{\text{l}}_{{\text{ss}}}}+{\text{ }}{{\text{H}}_{\text{2}}}{\text{O }}+{\text{ e}} \to {{\text{H}}_{{\text{ads}}}}+{\text{ OH}}$$

7 $${{\text{H}}_{{\text{ads}}}}+{\text{ }}{{\text{H}}_{\text{2}}}{\text{O}} \to {{\text{H}}_{\text{2}}}\,+\,{\text{O}}{{\text{H}}^ - }+{\text{ A}}{{\text{l}}_{{\text{ss}}}}$$

The HER at Al surface encountered due to the small activation overvoltage (b = 120 mV) for the slow discharge (H^+^+ e^−^→ H_ads_) rate determining step (RDS).

## Results and discussion

### Characterization of the electrocatalyst

**Fig. 5:**
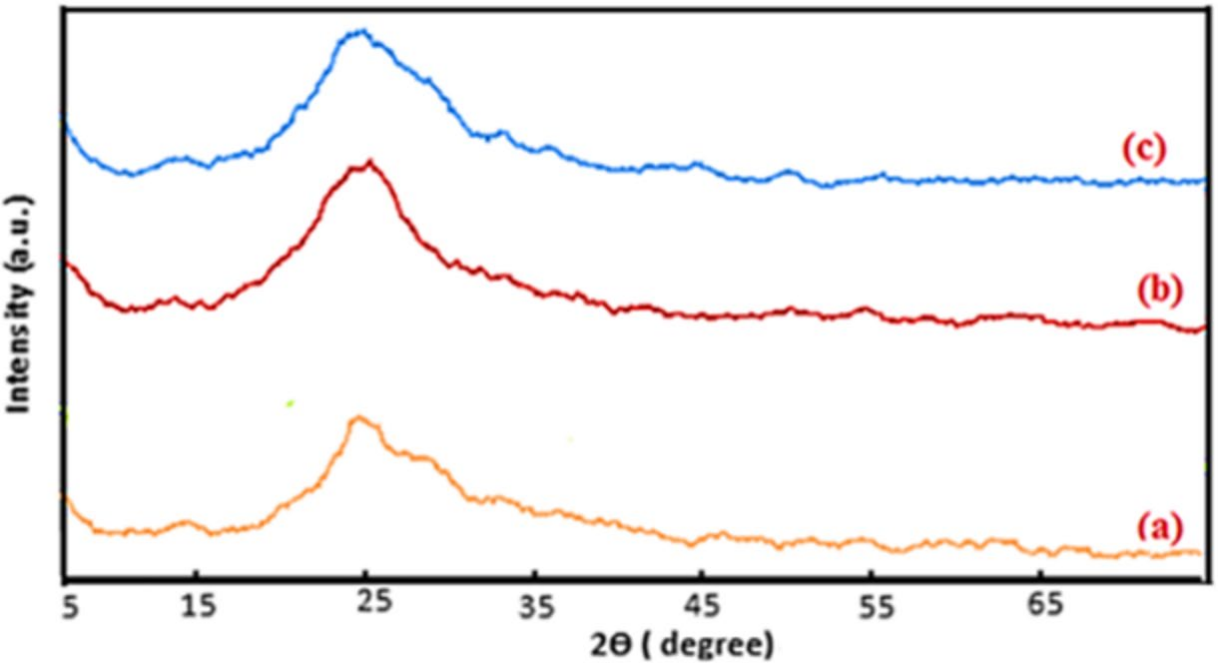
pXRD pattern: (a) NiO, (b) NiO-Fe_2_O_3_, (c) NiONC. The nearly same diffraction patterns with slightly modified crystallinity confirmed the formation of metallic matrix of NiO.

Figure 5 showed powder pXRD diffraction pattern confirmed compatible loaded Fe_2_O_3_ and NiONPs on graphite matrix.

Semicrystallinity of NiONC improved the catalytic activity.

The average crystal size of thin film of NiONC calculated using Eq. 8.

8 $$\:\text{C}\text{r}\text{y}\text{s}\text{t}\text{a}\text{l}\text{l}\text{i}\text{n}\text{e}\:\text{s}\text{i}\text{z}\text{e},\:\:\text{D}\:\left(\text{n}\text{m}\right)=\:\frac{\text{K}\:{\uplambda\:}}{{\upbeta\:}\:\text{c}\text{o}\text{s}\:{\uptheta\:}}$$

Where$\:\:{\upbeta\:}$ is the full width at half maxima (FWHM (Rad), Ө is the diffraction angle ( rad). The obtained results given in Table 2 indicate that the average particle size (PS) in the range 46.09–46.77 nm^19^.

**Table 2:** Crystalline size NiONC elctrocatalyst.

Ө^o^	Ө_rad_	FWHM	PS (nm)	Ө^o^	Ө_rad_	FWHM	PS (nm)
36	0.3958	0.0030	46.09	34	0.549	0.0029	46.77

**Fig. 6:**
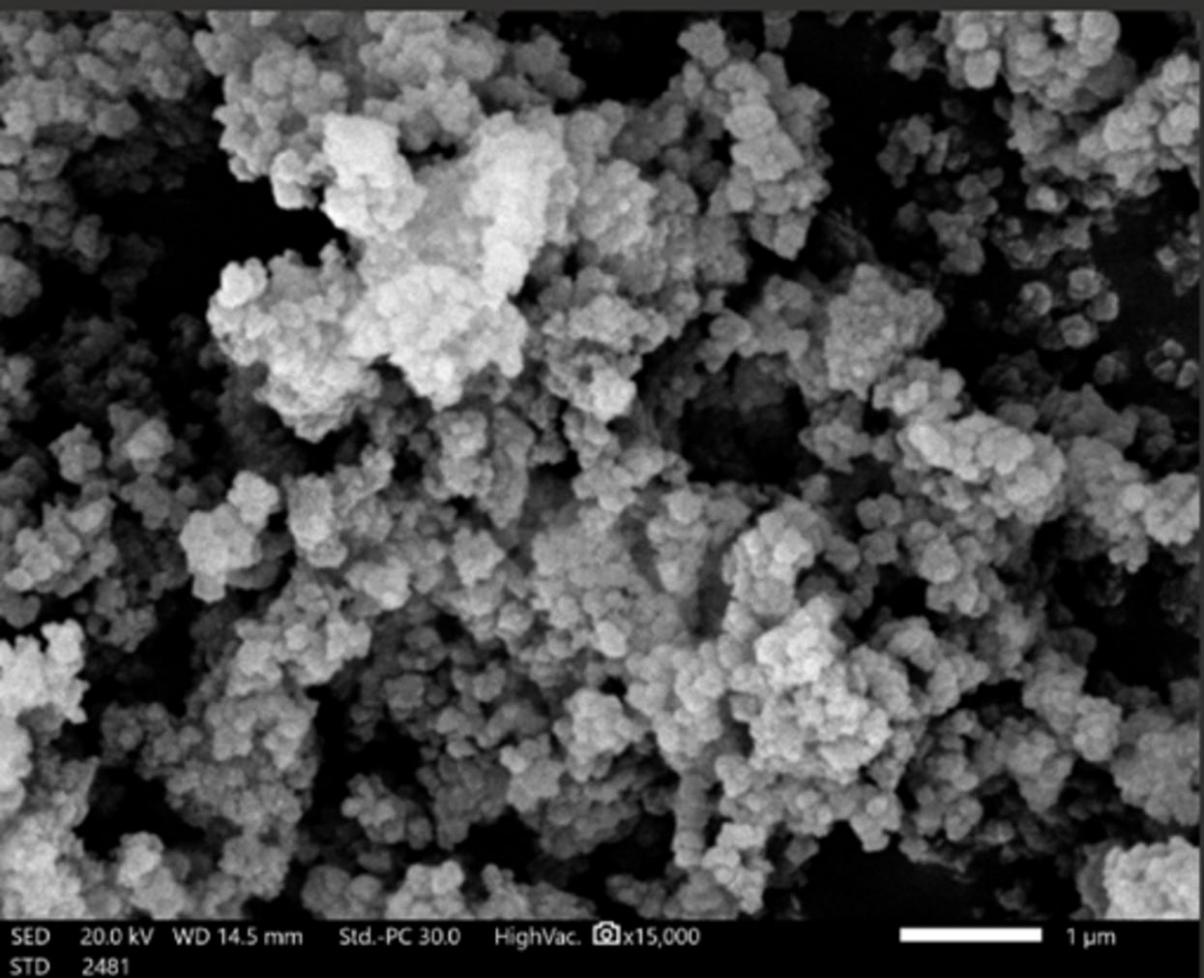
SEM micrograph of NiONPs (electrocatalyst).

**Fig. 7:**
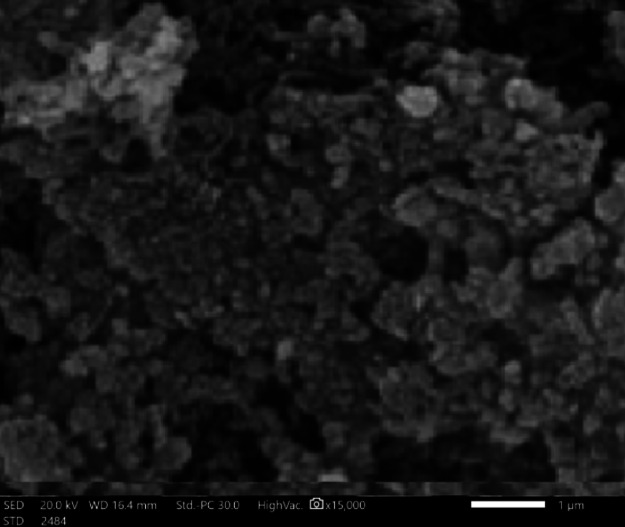
SEM micrograph of Fe_2_O_3_NPs (catalyst promoter).

**Fig. 8:**
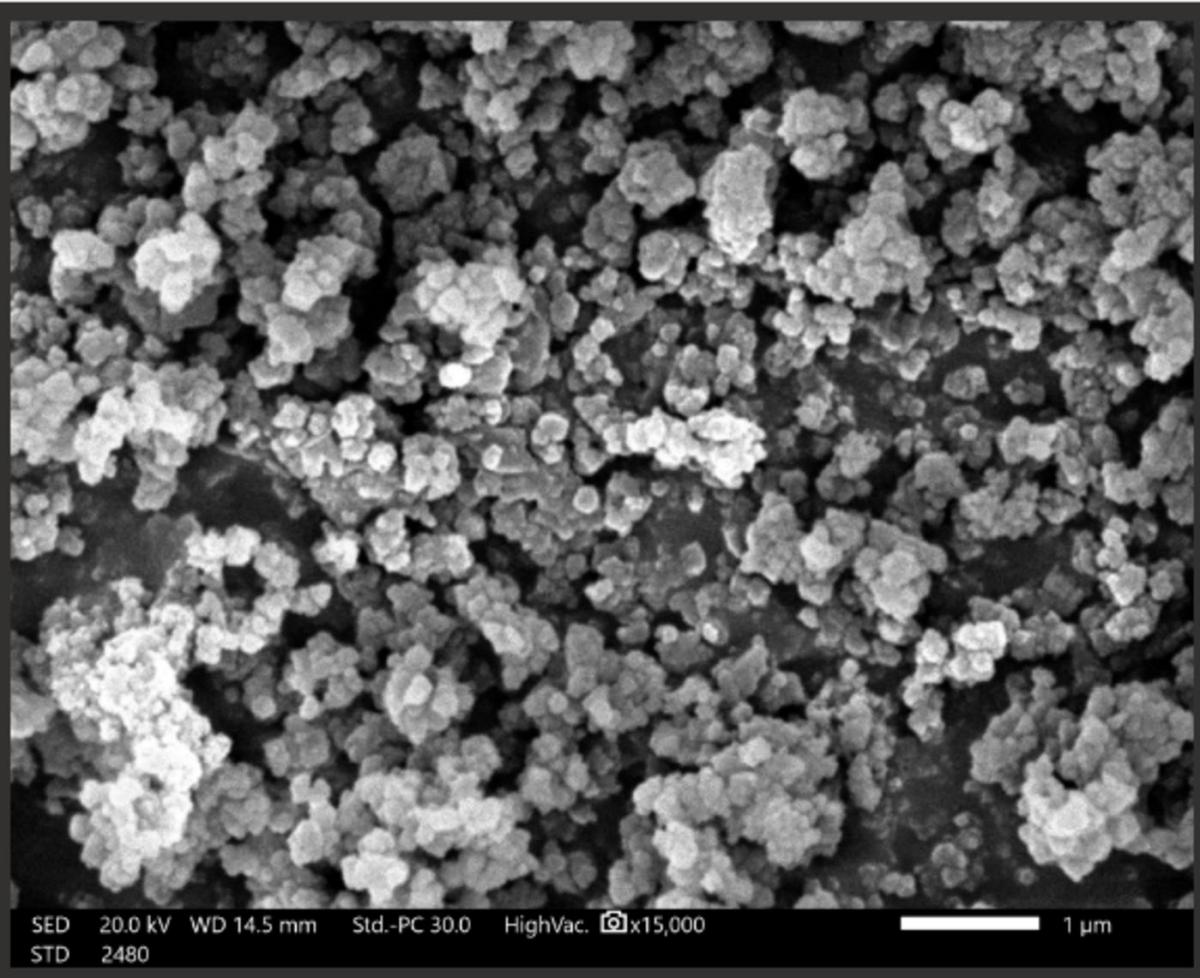
SEM micrograph of NiONC (electrocatalyst: S, F_3_O_4_-NiO@chitosan).

Figures 6, 7 and 8 confirmed modification of surface morphology of NiONPs by S, and Fe_2_O_3_ dopants. Nanocyrstalline TF have particle size less than100 nm. These TF have large surface area to volume ratio yielding quantum mechanical effects to ORR.

Since the crystal, size exceeded 30 nm. Hence, TFs exhibited a thermodynamically reversible phase transition to more stable phase. The larger the particle size, the rapid is the phase transformation. Nanocrystalline TFs containing confined electrons and having advanced electronic properties. The small particle size improved the interfacial interactions between the atoms constituents and improved the electronic properties^12,20^.

**Fig. 9:**
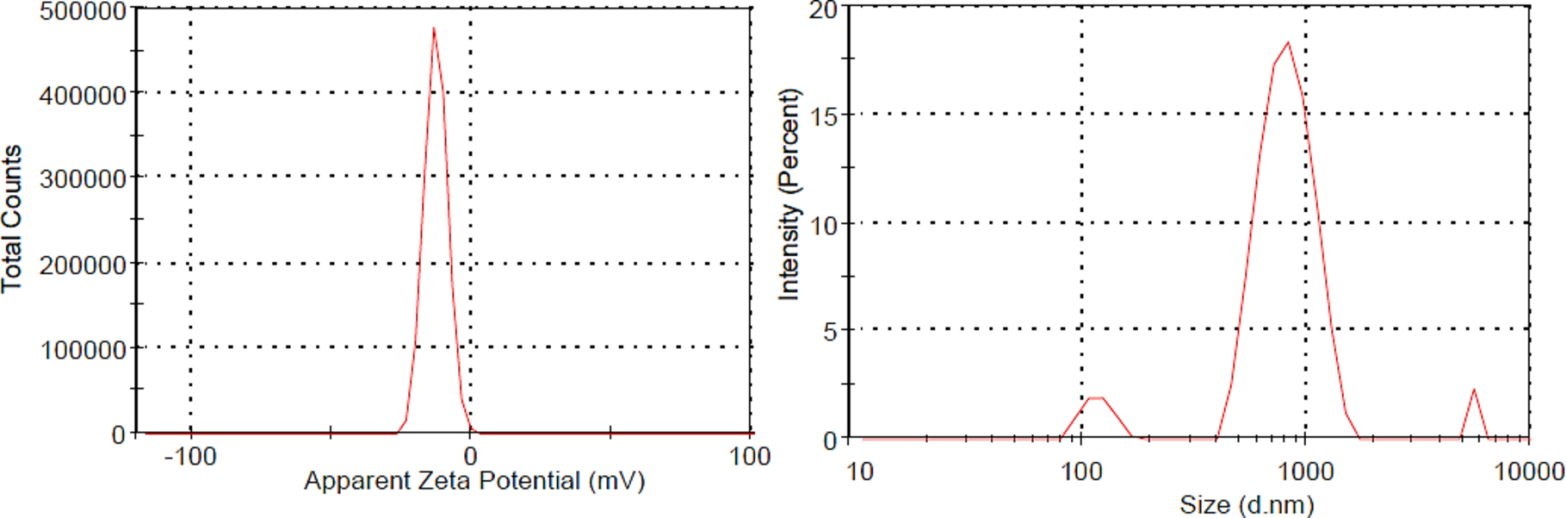
Zeta potential Particle size distribution of NiONC (electrocatalyst).

Figure 9 showed negative (-16.2 mV) zeta (ζ) potential reflect the stability of NiONC through the electrostatic repulsion forces between the NPs. ζ indicate NC are electrically stabilized and remain dispersed as suspension with no coagulation or flocculation^21^.

The particle size (PS) distribution of NiONC showed the average particles size distribution peaked at 1000 nm confirmed that both metallic NPs loaded and dispersed on the matrix of chitosan macromolecules. NPs on cross-linked chitosan matrix giving macromolecular dispersion. The polydispersity index 0.408 (less than 1.0 indicated mass average molecular weight is less than number average molecular weight) confirmed good dispersion. All these findings confirmed the perfect dispersion of electro catalyst NPs as colloidal system^22,23^.

**Fig. 10:**
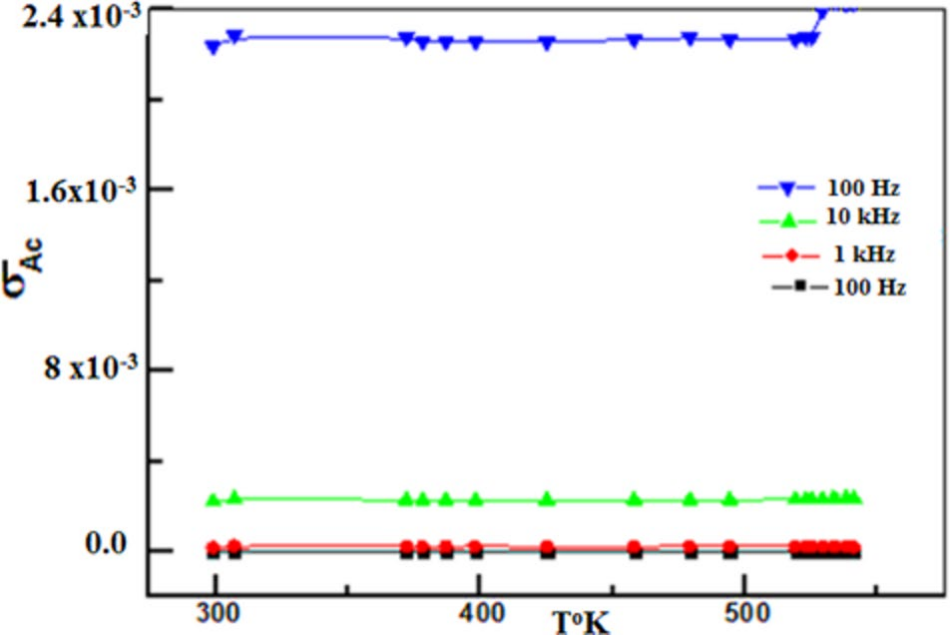
Variation AC conductivity of NiONC with frequency.

Figure 10 showed AC conductivity of NiONC increased with the applied frequency.

The high (σ_AC_) at high frequency confirmed rapid charge transfer process on the catalyst surface to the molecular oxygen facilitating ORR. Temperature range (30**°**C -220**°**C) slightly affected electrical resistivity ($\:\rho\:$) in the absence of magnetic field^23^.

**Fig. 11:**
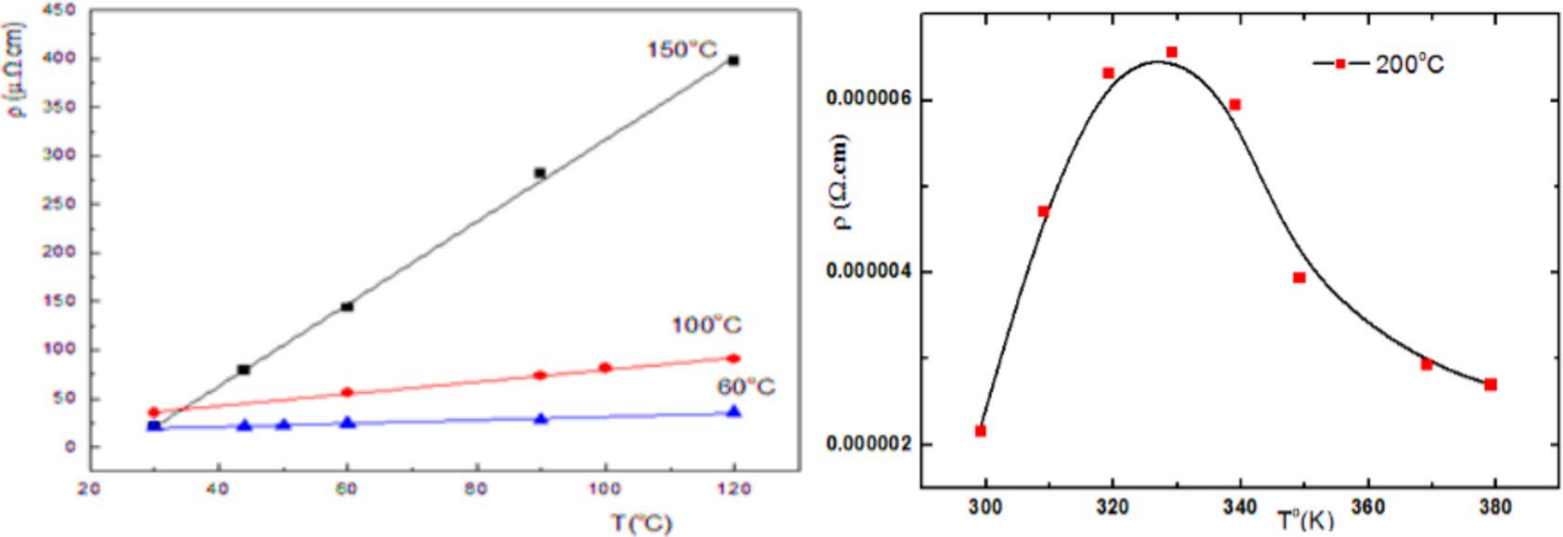
Relation between ($\:\rho\:$) and temperature (T) for NiNO annealed at different temperature.

Electrical resistivity ($\:\rho\:$) of NiONC sample annealed at 60 °C, 100 °C and 150 °C shown in Fig. 11 rising temperature to 120 °C, $\:\rho\:$ increased at high annealing temperature ( characterized metallic behavior of the prepared TF). For annealing at 200 °C,$\:\:\rho\:$ exhibited metallic character on heating up to 55 °C, and exhibited a semiconductor behavior (maximum) upon increasing temperature. The decrease of $\:\rho\:$ abovr 60 °C referred to the phase change, recrystallization and /or the formation of none stoichiometric oxide film^24^.

The unique behavior of NiONC air cathode annealed at 200^o^C suggested high annealing temperature improved electrode performance. Maximum $\:\rho\:$ at 60^o^C indicating phase transition. The sharp decrease of $\:\rho\:$ above 60^o^C indicated superconductivity. This NiONC cathode sample used in the evaluation of AFC^12,25^.

The electronic thermal conductivity (W/m. K) of ultra-TF (thickness less than 100 nm) calculated using Wiedermann-Franz (WF)^26^, Eq. 9.

9 $$\:\text{T}\text{h}\text{e}\text{r}\text{m}\text{a}\text{l}\:\text{c}\text{o}\text{n}\text{d}\text{u}\text{c}\text{t}\text{i}\text{v}\text{i}\text{t}\text{y}\:\left({k}_{e}\right)=\frac{\text{L}^\circ\:\:\text{T}}{\rho\:}$$

Where L° Lorenz number (2.44$\:\times\:$10^-8^ W.Ω.K^-2^) not used for bulk material and T is the absolute temperature (^o^K). The total conductivity of NiO is the sum of the thermal and the electronic conductivity. Thermal conductivity (ke) caused by phonons interaction and sound waves describing atoms vibration. Electronic conductivity is due to the kinetic energy of electrons and moving holes. The small value ke $\:1.1\times\:$10^− 7^ of NiONC indicated that the total conductivity of NiONC are solely due to electronic conductivity^26^.

**Fig. 12:**
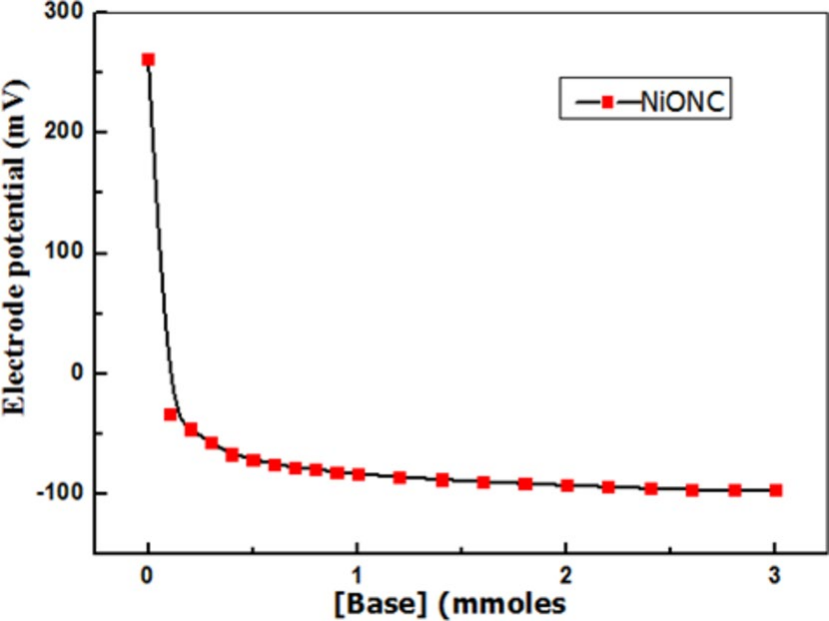
Potentiometric titration curve for NiONC electrocatalyst.

Figure 12 showed the variation of potentiometric E(mV) for NiONC calcined at 200^o^C with added volume of *n*-butylamine base. As acid sites neutralized electrode potential limited. Volume of base g^-1^catalyst equivalent to the surface acidity. At zero current potentiometry, potential of the indicator Pt electrode is a function of NiONC acidity. A cell constructed by placing indicator Pt electrode with saturated reference electrode (E_SCE_ 0.242 V) electrode in the titrated NiONC suspension. The e.m.f. of the cell measured after each base addition. Zero current in galvanometer confirmed balanced cell e.m.f. by the external applied potential^16^.

The initial electrode (E_i_ 100 mV) indicated strong acidic sites irreversibly bound to the entrapped base molecules. The shape of the sigmoidal potential-volume depends upon the equilibrium constant and the stoichiometry of the titration reaction. Potential of Pt electrode limited when all acid sites on NiONC occupied by the base^16^.

**Fig. 13:**
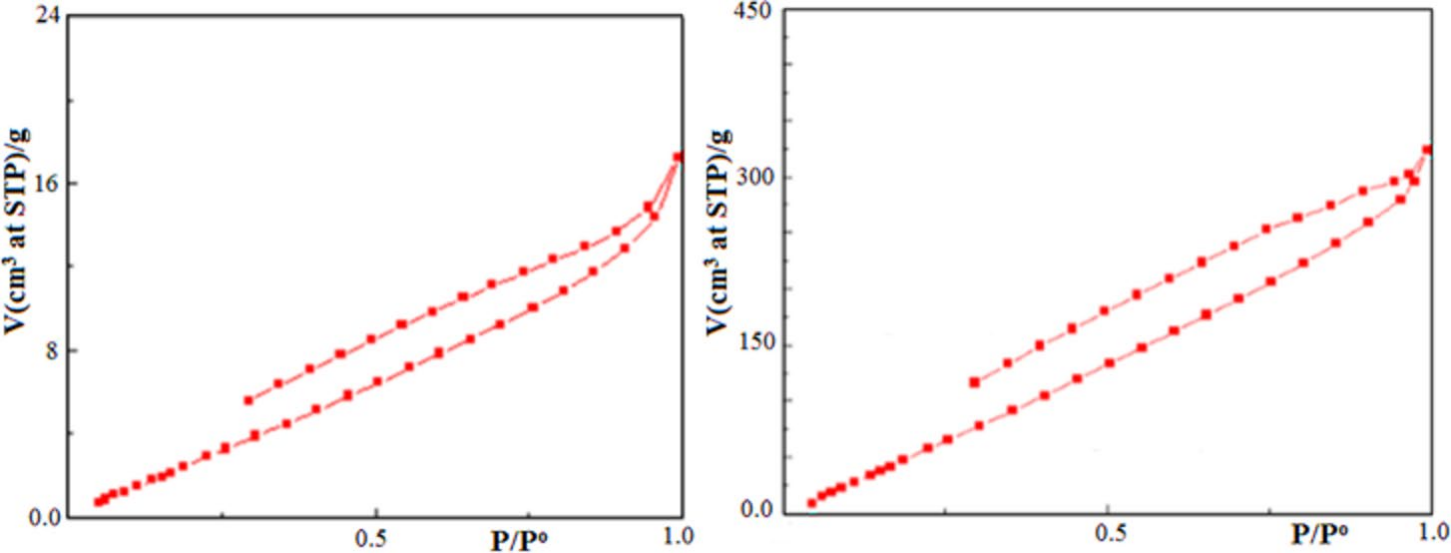
Adsorption-desorption isotherm for nitrogen on NiONC.

Textural properties of NiONC based on BET Theory of multilayer Nitrogen gas adsorption. Total volume of the micropores (widths smaller than 2 nm). N_2(g)_ adsorption-desorption isotherm curves, Fig. 13 followed type (IV) isotherm according to IUPAC classification indicating cathode surface contains micro- and meso pores. The hysteresis loops confirmed gas desorption (on lowering gas pressure) from narrow neck bottle like pores. The developed internal micro and mesopores pores increased adsorption capacities from 17.208 cm^3^g^− 1^ in activated graphite and 323.89 cm^3^g^− 1^ in NiONC. Higher adsorbed volume (confirmed larger numbers of microspores) than Pt or graphite cathode^27^.

For NiONC, volume of nitrogen for monolayer completion (Vm) was 347 cc (STP, g^-1^), Specific BET surface area (S_BET_) 70.23 m^2^g^-1^, adsorption constant (C) 47.11, mean pore diameter 3 nm, 9.73 nm for micro-and mesopores respectively. The total pore volume (V_T_) at relative pressure P/Po 0.99 equals 192.71 cm^3^g^-1^.

### Evaluation of the acidic AFC

**Fig. 14:**
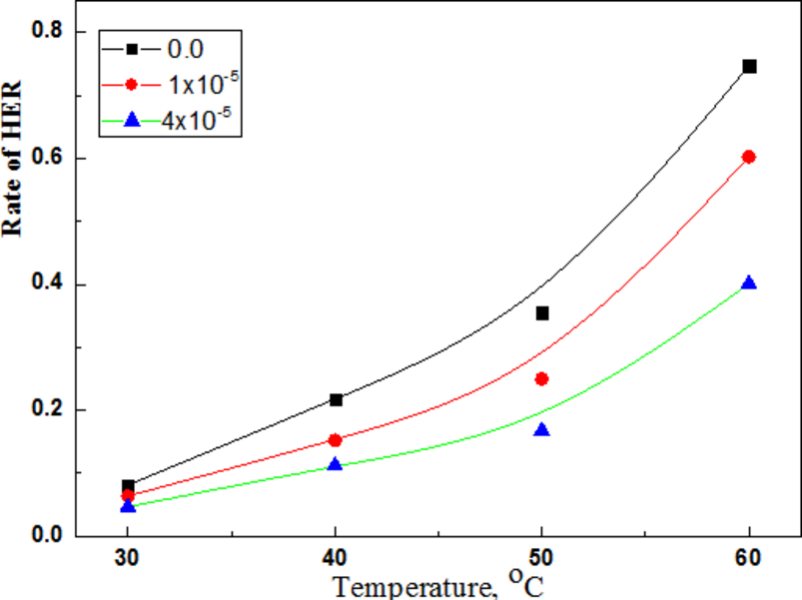
The effect antimony sulphate on rate HER for Al/0.1 M HCl at 30^o^C. Rate of HER (mLcm^− 2^min.^−1^) dramatically decreased by 4 × 10^− 5^M Sb_2_(SO_4_)_3_ at all temperatures.

Figure 14 showed lowering of hydrogen evolution reaction by the antimony sulphate.

The hydrogen poison Sb_2_(SO_4_)_3_ decreased rate of HER up to 60^o^C operation temperature of AFC. These antimony ions specifically retard HER and very effective in acid solutions but are ineffective in environments where other reduction processes such as oxygen reduction are the controlling cathodic reactions. Twenty-five atm. H_2_ poisoned on using 4 × 10^-5^ M Sb_2_(SO_4_)_3_ compared to only 3.0 atm. H_2(g)_ at 25 °C in 20 h using the same concentration of toxic and carcinogenic chromate solution as CI for Al^28^.

Antimony ions diffused through surface Al_2_O_3_ film, formed resistive transitory compounds in Al_2_O_3_ that increased charge transfer resistance (Rct) on Al surface and retarded HER and Al corrosion^29,30^.

**Fig. 15:**
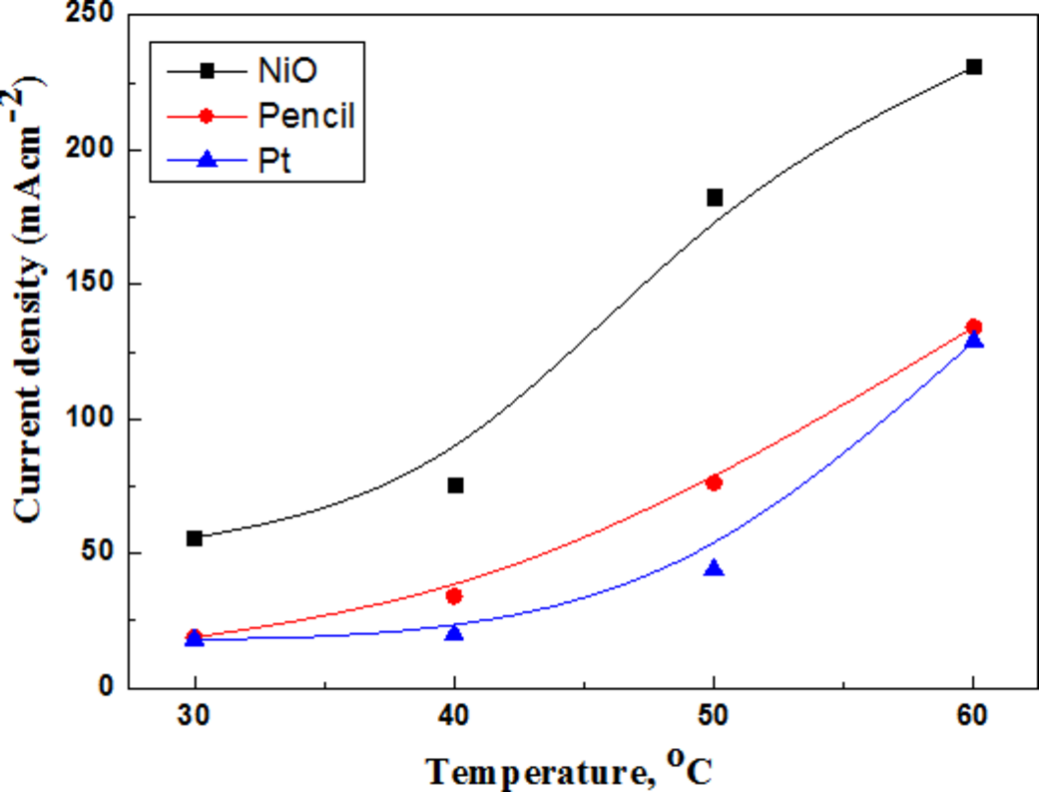
Variation of discharging current density with temperature.

Current density obtained during discharging represented in Fig. 15.

Data in Table 3 for the acidic AFC cell discharged at 40 °C, 1.44 V, 1.52 V for Pt and graphite cathode respectively. Current densities increased from 11.77 mAcm^-2^ to 10.20 to mAcm^-2^ (on replacing graphite by Pt cathode). The cell power energy calculated using Eq. 10^12^.

10 $${\text{Energy power }}\left( {{\text{P}},{\text{ kWhk}}{{\text{g}}^{ - {\text{1}}}}} \right)\,=\,{\text{IV }}*0.0{\text{6}}$$

**Table 3:** Electrochemical parameters of AFC of AFC.

Air cathode	[Sb_2_SO_3_]	E_Cell_ (V)	Current density A.m^− 2^		Power (kWhkg^− 1^
Pt	1 × 10^− 5^	1.44	118		10.20
Graphite	4 × 10^− 5^	1.52	129		11.77
NiONC	0.1	2.4	240		30.30

High [Sb_2_SO_3_] increased power density. Dilute solution 0.1 M HCl is less economic costs and more safe than alkali (uncontrolled rate of HER, large concentration decreased current density of Al and enhanced Al(OH)_3_ gelation by hydroxyl ions.

Power density obtained in this study (30.30 kWhkg^− 1^) is much higher than 0.360 kW h dm^− 3^ obtained from Al/H_2_O_2_ FC, energy density 0.290 kW h dm^− 3^ from Al AgO FC. While Al/air semi FC with H_2_O_2_ co-generation yield power (0.017 kWcm^− 2^) and maximum power 10.44mW cm^− 2^.

Cell reaction of Al/0.1 M HCl (pH 1) represented as by Eq. 11:

11 $${\text{Active dissolution}}:{\text{ Al}} \rightleftharpoons {\text{A}}{{\text{l}}^{{\text{3}}+}}+{\text{ 3}}{{\text{e}}^ - }$$

Oxidation of every Al atom liberated 3es^−^. The high chemical equivalent of Al hindered and cell efficiency is usually less than unity due to chaotic problems that declined both the cell potential and the closed current density under load (discharging). Cell parameters AFC in uninhibited and inhibited electrolyte collected in Table 4.

**Table 4:** Electrical parameters for AFC/different aqueous electrolytes.

Electrolyte	E_cell_ (V)	I (A. m^− 2^)	kW h kg^− 1^
0.1 M HCl	1.537	5	4.61
0.1 M HCl+ (Sb_2_(SO_4_)_3_	1.738	12	5.31

Cell potential of AFC increased by Sb_2_(SO_4_)_3_ (above two folds higher current density). Advantageous micro-AFC (Al/HCl, 5.31 kWh kg^−^1 can provide constant power.

The redox reactions of Al/HCl heterogonous system represented by Eqs. 12, 13.

12 $${\text{Al}} \rightleftharpoons {\text{A}}{{\text{l}}^{{\text{3}}+}}+{\text{ 3}}{{\text{e}}^ - }$$

13 $${\text{3}}{{\text{H}}^+}+{\text{ 3}}{{\text{e}}^ - } \to {\text{ 3}}/{\text{2 }}{{\text{H}}_{{\text{2}}({\text{g}})}}$$

Overall discharging reaction: Al + 3HCl → AlCl_3_ + 3/2 H_2(g)_.

The power density of the cell calculated from I-V curves for AFC connected to 10kOhm load resistance. The data collected in Table 5.

**Table 5:** Discharging parameters of AAFC in different electrolytes.

Electrolyte	E_cell_ (V)	I, (A.m^− 2^)	kWhkg^− 1^
0.1 M HCl/Pt	0.7332	16	0.7039
0.1 M HCl/NiONC	1.2	24	28.8

The strong high electrically conductive HCl electrolyte increased current density of AFC. Al(OH)_3_ gel formation is less encountered than in NaOH as the predominant species are H^+^. However, HER should be inhibited, Eq. 14

14 $${\text{AlC}}{{\text{l}}_{\text{3}}}\,+\,{\text{C}}{{\text{l}}^--}~\; \rightleftharpoons \;{\text{AlC}}{{\text{l}}^{ - \,}}_{{\text{4}}}$$

Power density (P, Watt) calculated from the integrated area of I-V curve. The energy density calculated using Eq. 15.

15 $${\text{Energy density }}\left( {\text{E}} \right)\,=\,{\text{P}}*{\text{t}}\,=\,{\text{I}}*{\text{t}}/{\text{F}} \times {\text{V}}$$

Where I, V, t, F are the current (A), solution volume (m^3^), time (min.), F: Faraday’s constant (96 500 C mol^− 1^).

For AFC, the current densities (*J*) 10–20 A m^− 2^ is equivalent to E (25 kW h kg^− 1^), J (20–60 A m^− 2^) equivalent to 80 kWh kg ^− 1^.

Ions migration increased discharge capacity of AFC: During discharging, number of electrons (es) delivered from AFC (current flow). The cell capacity (Ahg^− 1^) equals the product of current (I) x time of discharge (t).

The capacity WhV^− 1^^− 1^ min.^−1^ equals the amount of energy delivered from cell to the loading during vehicle operation. Discharge voltage curve should remain relatively constant until most of capacity discharged then drop off sharply. Area of relatively constant voltage (voltage plateau) at which the voltage rapidly declined (curve knee) extended to higher potential on inhibition of HER by antimony ion that increase capacity of AFC and elongate discharge time.

Table 6 confirmed improved discharging performance of AFC: (Al/HCl/NiONC). Potential-time curve recorded using conventional high sensitive-wide range detector galvanometer.

**Table 6:** Capacities AFC discharge at constant uniform current (15 mA cm^− 2^).

Solution	*R*, mL min^− 1^ cm^− 2^	Time discharge, min.	Capacity Ahg^− 1^
0.1 M HCl	0.099	93	1.450
0.1 M HCl + 4 × 10^− 5^M Sb_3_ ions	0.065	140	5.031

NiONC is n-type semiconductor contain excess Ni(II) ions and conducted electricity by means of negative charge carriers (namely the electrons). These delocalized electrons present to maintain electrical neutrality. The presence of excess metal cations achieved anion non-stoichiometric vacancies such as oxygen (O_2_ ^− 2^) from Fe_2_O_3_.

Similarly, in P-type semiconductor oxides have excess anions metal ion deficiency in the lattice conduct by means of positive holes which are essentially cations have an extra positive charge due to missing electrons, e.g. Ni^+ 3^ (Ni^+ 2^-e^−^). The anion excess achieved by interstitial anions or cation vacancies (example FeO, NiO). Microstructure of n- and p- type oxides. Each lattice drawn electrically neutral with respect to the defects present.

Electrical conductivity within the oxides NPs controlled by electrons (es) flow while oxidation rate or ions flow is the ionic conduction. In p-semiconductor oxides, es flow occurs *via* positive holes (es deficient sites) while ionic conductivity (and hence oxidation) occurs by cations diffusion *via* vacant cations sites. In n-semiconductor, es flow occurs by movement of free es while ionic conductivity (oxidation) *via* diffusion of O^− 2^ ions (through vacant anions sites) or interstitial cations.

General pitfalls for commercialization acidic AFC: commercial Al is suitable anode because of high electrical conductivity and mechanical strength and the low CR. Anode thickness designed for long service since corrosion is a penetrating action, it is necessary to make allowances for the reduction in anode thickness to give at least ten years’ service life. Weld electrical conductors to the electrodes rather than rivets joints to avoid crevice corrosion. Design cell tank for easy draining and cleaning. Tank bottoms of AFC slopped toward drain holes, so liquids cannot collect after emptied. Design for easy replacement of consumed Al anode.

## Conclusion

Simple low cost AFC in slightly acidic strong electrolyte 0.1 M HCl designed. The electrodes materials were commercial Al anode (under de-aerated conditions) and NiONC an efficient porous air cathode with permanent template pores. NiONC is a promising electrocatalyst for the slow oxygen reduction reaction. Sb efficiently poisoned the interference HER. Irreversible adsorption of antimony ad-atom on Al surface enhanced by sulphur atom. High power density AFC enabled application to electric vehicle than biofuel and solar power.

## Data Availability

“All data generated or analysed during this study are included in this published article”.

## Abbreviations

AFC: Aluminum Fuel Cell
I: Electrical current
Al: Aluminum metal
ORR: Oxygen reduction reaction
Ah/g: Ampere hour per gram
Wh/g: Watt hour per gram
DO: Dissolved Oxygen
HER: Hydrogen evolution reaction
SHE: Standard Hydrogen Electrode
CR: Corrosion rate
IE: Inhibition efficiency
DO: Dissolved Oxygen
